# Mesenchymal Stem/Stromal-Like Cells from Diploid and Triploid Human Embryonic Stem Cells Display Different Gene Expression Profiles

**DOI:** 10.29252/ibj.25.2.99

**Published:** 2020-11-11

**Authors:** Mahdieh Javidpou, Seyed-Morteza Seifati, Ehsan Farashahi-Yazd, Fatemeh Hajizadeh-Tafti, Jalal Golzadeh, Fatemeh Akyash, Behrouz Aflatoonian

**Affiliations:** 1 Stem Cell Biology Research Center, Yazd Reproductive Sciences Institute, Shahid Sadoughi University of Medical Sciences, Yazd, Iran;; 2 Medical Biotechnology Research Center, Ashkezar Branch, Islamic Azad University, Ashkezar, Yazd, Iran;; 3 Department of Reproductive Biology, School of Medicine, Shahid Sadoughi University of Medical Sciences, Yazd, Iran;; 4 Department of Advanced Medical Sciences and Technologies, School of Paramedicine, Shahid Sadoughi University of Medical Sciences, Yazd, Iran;; 5 Medical Nanotechnology and Tissue Engineering Research Center, Yazd Reproductive Sciences Institute, Shahid Sadoughi University of Medical Sciences, Yazd, Iran

**Keywords:** Human embryonic stem cells, Mesenchymal stem/stromal cells, Regenerative medicine

## Abstract

**Background:**

hESCs-MSCs open a new insight into future cell therapy applications, due to their unique characteristics, including immunomodulatory features, proliferation, and differentiation.

**Methods:**

Herein, hESCs-MSCs were characterized by IF technique with CD105 and FIBRONECTIN as markers and *FIBRONECTIN*, *VIMENTIN*, *CD10*, *CD105*, and *CD14* genes using RT-PCR technique. FACS was performed for CD44, CD73, CD90, and CD105 markers. Moreover, these fibroblast-like cells, due to multipotent characteristics, differentiated to the osteoblast.

**Results:**

MSCs were derived from diploid and triploid hESC lines using sequential 3D and 2D cultures and characterized with the specific markers. IF showed the expression of FIBRONECTIN and CD105 in hESCs-MSCs. Flow cytometry data indicated no significant difference in the expression of MSC markers after 6 and 13 passages. Interestingly, gene expression profiles revealed slight differences between MSCs from diploid and triploid hESCs. The hESCs-MSCs displayed osteogenic differentiation capacity, which was confirmed by Alizarin red staining.

**Conclusion:**

Our findings reveal that both diploid and triploid hESC lines are capable of forming MSCs; however, there are some differences in their gene expression profiles. Generation of MSCs from hESCs, as a non-invasive procedure in large scale, will lend itself for the future cell-based therapeutic applications.

## INTRODUCTION

Since the first report on hESCs derivation^[^^1^^]^, hESCs have hitherto been derived from fresh and frozen embryos at different stages of development using various techniques^[^^2^^,^^3^^]^. Interestingly, hESCs derived from 3PN human zygotes display both normal diploid and abnormal triploid karyotypes^[^^4^^]^. It has been shown that triploid hESCs derived from 3PN zygotes have similar pluripotency characteristics to diploid cell lines^[^^5^^]^. Besides, the biological features and differentiation potential of normal and abnormal hESCs lines have been confirmed to be the same^[^^6^^]^. 

Recently, scientists have focused on the generation of hESCs-MSCs and their use in regenerative medicine^[^^7^^]^. Data from several research groups have indicated that these hESC-MSCs show similar characteristics to bone marrow-derived MSCs^[^^8^^-^^10^^]^. They applied embryoid body formation (3D) or monolayer (2D) culture methods for derivation of MSCs form hESCs^[^^8^^-^^10^^]^. However, the invasive procedures and the limited quantities of adult tissue-derived MSCs, together with some technical challenges in purification and enrichments of these cells, hamper the extensive application of these type of cells in clinical use. In this sense, the hESCs-MSCs with large proliferation capacity and non-invasive derivation procedure can provide clinical grade source of MSCs^[^^7^^]^. 

Various methods have already been utilized for generating MSCs from hESCs^[^^8^^-^^10^^]^. The EB formation is a novel approach for the generation of hESCs-MSCs^[^^9^^]^. Recently, Yan and co-workers^[^^10^^]^ have used spheroids as a 3D culture, and concluded that it is an affordable, scalable and simple method for derivation of high-quality MSC from hESCs. In comprised adult MSCs, hESCs-MSCs, as a non-invasive derivation technique and simple expansion, can be used as suitable source of MSC cell banking for clinical applications^[^^7^^-^^10^^]^. Herein, for the first time, we used sequential 3D and 2D culture for derivation of MSCs from both diploid (Yazd2) and triploid (Yazd3) hESCs. However, some differences were observed between the gene expression profiles of the MSCs derived from diploid and triploid hESCs were observed.

## MATERIALS AND METHODS

Chemicals were purchased from Sigma Aldrich (Poole, UK). Culture media and supplements were obtained from Invitrogen, Gibco (UK), unless otherwise stated.

**Cell lines**

The hESC lines used in this study were Yazd2 as diploid (46,XY) and Yazd3 as triploid (69,XXY), which were produced by the Stem Cell Biology Research Center based in Yazd Reproductive Sciences Institute, Yazd, Iran^[^^3^^,^^11^^]^. 

**Generation of hESC-MSCs**

Sequential 3D and 2D culture using EB formation^[^^3^^]^ and subsequent monolayer culture were applied as the differentiation method for derivation of MSCs from hESCs. In the first step of differentiation, hESCs were cultured into EB medium for eight days, and following that, for monolayer culture, EBs were transferred into the tissue culture flasks (Falcon, USA) containing DMEM supplemented with 10% FBS. The hESCs-MSCs were passaged enzymatically as a single cell suspension. This procedure was repeated until the cell population became phenotypically homogeneous with fibroblast-like cell morphology. The MSCs were passaged and expanded in the tissue culture flasks and incubated in the humidified atmosphere at 37 °C and 5% CO_2_, while cells population was above 90% confluency. 

**IF localization for cell markers**

For IF staining, after three times washing of the cells with PBS for five minutes, fixation of the cells was performed using 4% paraformaldehyde solution at room temperature for 15 minutes. Samples were washed twice in PBS for five minutes. Permeabilization of the cells was performed with 0.1% Triton X-100 for 10 minutes. Nonspecific binding of the primary antibodies was prevented by incubating the cells in PBS with 5% FBS (as the blocking solution) for 30 minutes before 24-hour incubation with primary antibodies (CD105 [ab1141] and FIBRONECTIN [ab6328]) at 4 °C. Incubation with secondary antibodies was carried out for one hour at 37 °C. Finally, the fluorescence microscopy was used for the evaluation of samples with appropriate excitation optics on an inverted microscope (Olympus IX-71)^[^^12^^]^. Negative controls were incubated only with the secondary antibody (anti-mouse IgG [FITC]; ab6785).

**RNA extraction, cDNA synthesis, and RT-PCR**

After cell collection, total RNA extraction was conducted by TRI Reagent according to the manufacturer’s instructions. After treatment of the extracted RNA by DNase I, cDNA was synthesized using the Thermo Fisher Scientific (USA) kit based on the manufacturer’s protocol. cDNA were then subjected to PCR amplification in a 20-µl final reaction volume including 1 µl of cDNA, 0.5 µl of each primer (20 mM), and 6 µl of dH_2_O, and 12 µl of Taq DNA Polymerase Master Mix RED (Ampliqon, USA). The *β2M* gene was used as the internal control, and target genes, including *FIBRONECTIN*, *VIMENTIN*, *CD10*, *CD105*, and *CD14* were evaluated. Amplifications were carried out in 35 cycles as follow: initial denaturation step at 94 °C for 5 minutes, denaturation step at 94 °C for 30 seconds, annealing step at 58 °C (*FIBRONECTIN*, *VIMENTIN*, *CD10*, *CD14* and *β2M*) or 60 °C (*CD105*) for 30 seconds, extension for 30 seconds at 72 °C and a final extension of 5 minutes at 72 °C. PCR products were detected on 2% agarose gels electrophoresis. The summary for primers used in this study is listed in Table 1.

**Osteogenic** **differentiation of hESCs-MSCs**

For osteogenic differentiation, 3 × 10^3^cells/cm^2^ were cultured using DMEM + 10% FBS. At approximately 70% confluency, the medium was replaced with an osteogenic medium containing DMEM supplemented with 50 mg/mL of ascorbic acid 2-phosphate, 10 nM of dexamethasone, and 10 mM of β-glycerophosphate. The medium was changed every three days for 21 days. To detect the mineralization of the matrix, cultured cells were washed twice using PBS, fixed in paraformaldehyde 4% for 30 minutes and stained with 40 mM of Alizarin red for 15 minutes^[^^12^^]^. 

**Characterization of hESCs-MSCs using flow cytometry**

After dissociation of Yazd2-MSCs from different passage numbers (P6 and P13) using 0.25% Trypsin-EDTA enzyme, cells were washed with PBS containing 0.5% FBS. CD44, CD73, CD90, and CD105 (BD Bioscience, San Jose, CA, USA) were used as primary antibodies for 30 minutes and stored at 4 °C. In the following, the cells were incubated by appropriate fluorescent-conjugated secondary antibodies in the dark at 4 °C for 60 minutes. Samples were analyzed on a BD FACS Calibur (BD Biosciences, USA). Data analysis was performed using FlowJo 7.6 software. 

**Western blotting**

Western blotting technique was performed for CD10 and VIMENTIN according to the standard protocols explained elsewhere^[^^13^^]^.

**Statistical analysis**

Differences in normalized expression values between samples were assessed using the unpaired t-test. Experimental data were expressed as mean ± SD. Statistical differences were set at *p* < 0.05. 

## RESULTS

**Derivation of the hESCs-MSCs**

**Fig. 1:**
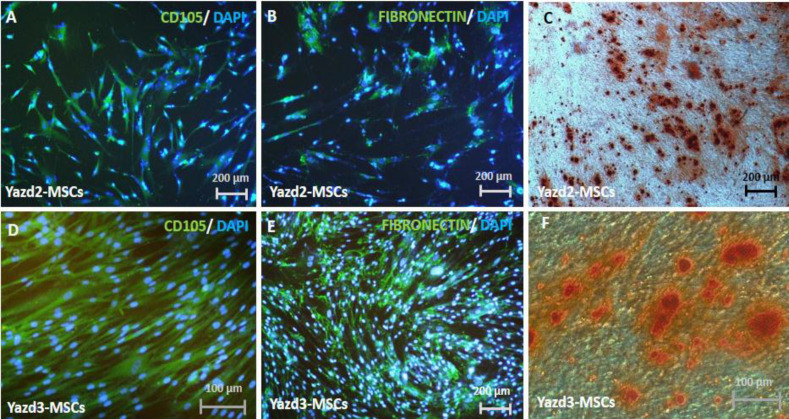
Characterization of the (A) Yazd2-MSCs and (D) Yazd3-MSCs with CD105/DAPI and (B)Yazd2-MSCs and (E) Yazd3-MSCs with FIBRONECTIN/DAPI; (C) osteogenic differentiation of Yazd2-MSCs identified with Alizarin red staining;; (F) Alizarin red staining for identifying the osteogenic differentiation of Yazd3-MSCs

MSCs were derived from diploid (Yazd2) and triploid (Yazd3) hESC lines using sequential 3D (EB formation) and 2D (monolayer) culture. At first, their spindle shape indicated the formation of MSCs, which were passaged using enzymatic treatment following expansion (Fig. 1A, 1B, 1D, and 1E).

**Characterization of the hESCs-MSCs using IF**

MSCs derived from both diploid and triploid hESC lines expressed MSC markers CD105 (Fig. 1A and 1D) and FIBRONECTIN (Fig. 1B and 1E).

**Gene expression analysis**

**Fig. 2:**
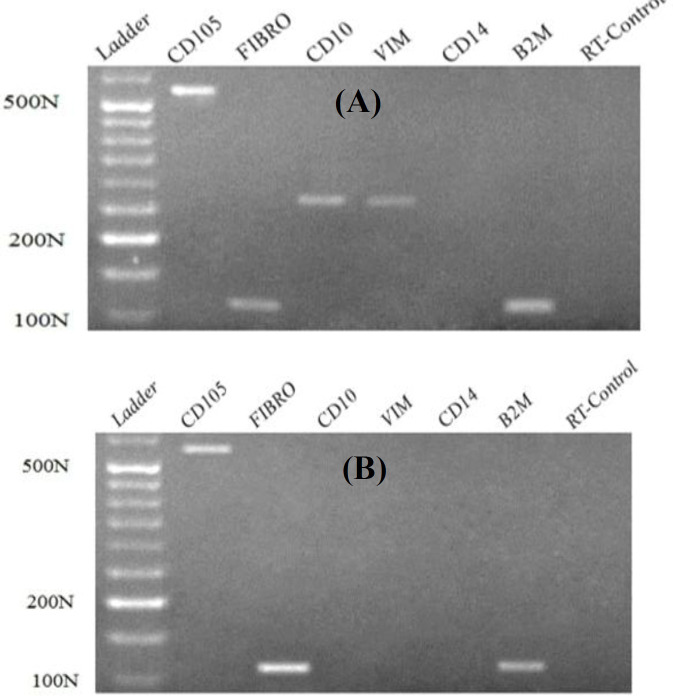
Reverse-transcription PCR for Yazd2-MSCs revealed the expression of* CD105*, *FIBRONECTIN*,* VIMENTIN*, and *CD10* (A). Also, expression of *CD105* and *FIBRONECTIN *was detected in Yazd3-MSCs (B), which did not express *VIMENTIN *and *CD10*. *CD14 *was not expressed in none of the cells

Among the candidate genes, *CD105*,* CD10*,* FIBRONECTIN*, and* VIMENTIN* were expressed in Yazd2-MSCs (Fig. 2A). Meanwhile, Yazd3-MSCs expressed only *CD105* and *FIBRONECTIN* (Fig. 2B). Cells did not express *CD14* (specific hematopoietic marker; Fig. 2). 

**Table 1:** List of primers used for RT-PCR

**Gene**	**Forward primer**	**Reverse primer**	**Annealing temperature** ** (°C)**	**Product ** **Size** **(bp)**
*CD105*	5’CTTGGCCTACAATTCCAGCC3’	5’CTTGAGGTGTGTCTGGGAGC3’	542	60
*CD10*	5’GGCACCAGAAGAACAGTAGG3’	5’ATCTCAGCATCAGTCAAAGC3’	269	58
*FIBRONECTIN*	5’AGGAAGCCGAGGTTTTAACTG3’	5’AGGACGCTCATAAGTGTCACC3’	106	58
*VIMENTIN*	5’TCTATCTTGCGCTCCTGAAAAACT3’	5’AAACTTTCCCTCCCTGAACCTGAG3’	270	58
*CD14*	5’CACACTCGCCTGCCTTTTCC3’	5’GATTCCCGTCCAGTGTCAGG3’	450	58
*β2M*	5’AGATGAGTATGCCTGCCGTG3’	5’TGCGGCATCTTCAAACCTC3’	106	58

**Osteogenic differentiation potential of diploid and triploid hESCs-MSCs**

After a 21-day culture of hESCs-MSCs under osteogenesis condition, mineralized matrix was detected using Alizarin red staining to confirm the osteogenic differentiation capability of the cells (Fig. 1C and 1F).

**Western blotting analysis**

**Fig. 3:**
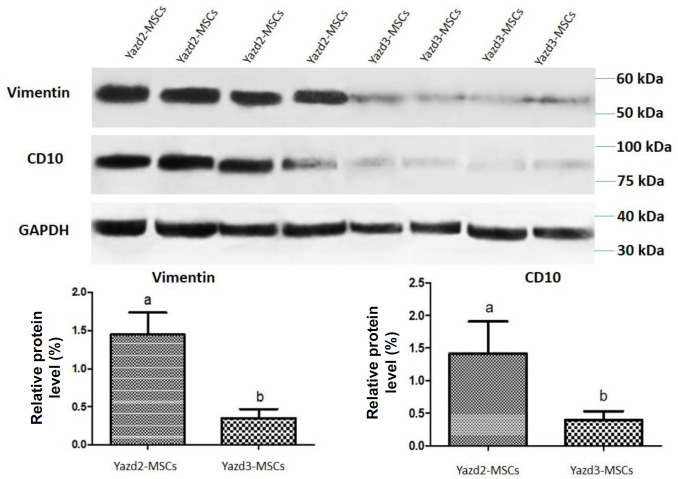
Western blot analysis of VIMENTIN and CD10 for Yazd2-MSCs and Yazd3-MSCs. Data indicate significant differences for VIMENTIN expression between Yazd2-MSCs (1.449 ± 0.1460) and Yazd3-MSCs (0.3517 ± 0.06069). Also, a higher expression level of CD10 was observed in Yazd2-MSCs (1.417 ± 0.2454) in comparison with Yazd3-MSCs (0.3986 ± 0.06506). “a” and “b” indicate the expression of *VIMENTIN* and *CD10* in Yazd2-MSCs and Yazd3-MSCs, respectively. Also, “a” in comprised “b” indicates the significant differences of Yazd2-MSCs with Yazd3-MSCs

Western blot analysis revealed higher protein expression level of VIMENTIN (1.449 ± 0.1460) and CD10 expression (1.417 ± 0.2454) within Yazd2-MSCs in comparison with their expression within Yazd3-MSCs (VIMENTIN: 0.3517 ± 0.06069; CD10: 0.3986 ± 0.06506; Fig. 3; *p* < 0.05). 

**Flow cytometry analysis in different passage numbers**

**Fig. 4:**
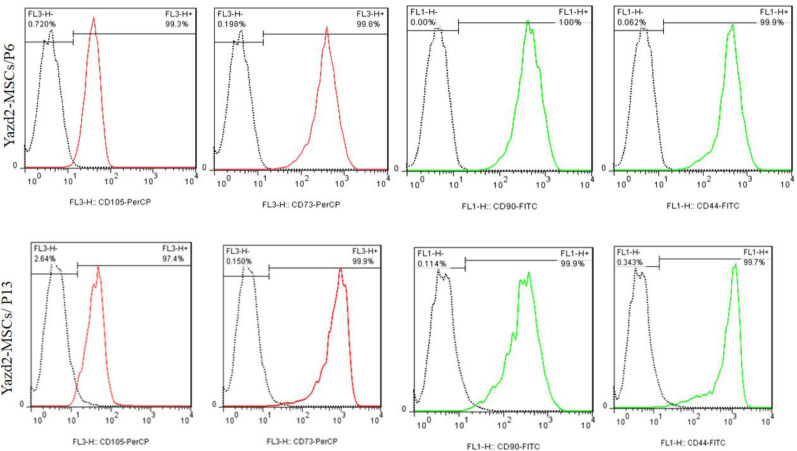
Characterization of Yazd2-MSCs using flow cytometry in different passage numbers (P6 and P13) with CD105, CD73, CD90, and CD44

Expression of MSC markers, including CD105, CD90, CD73, and CD44 in different passage numbers (P6 and P13) of Yazd2-MSCs was assessed using flow cytometry. The results indicated high levels of MSCs markers expression in both passage numbers (Fig. 4). 

## DISCUSSION

Human MSCs, due to immunomodulatory potential, are the best option for the treatment of autoimmune, degenerative diseases^[^^10^^]^. The main challenge of MSCs derivation from adult tissues in comparison with hESCs-MSCs is the limited expanded number of this derived cells using 2D culture system^[^^14^^]^. However, human pluripotent stem cells can be used as an unlimited source for derivation of MSCs^[^^15^^,^^16^^]^ with 3D^[^^10^^]^ and 2D^[^^8^^]^ culture system. 

Our data confirm that both diploid (Yazd2) and triploid (Yazd3) hESCs can form MSCs in culture similar to previous reports^[^^11^^-^^13^^]^. In sum, MSCs from normal (diploid; Yazd2) and abnormal (triploid; Yazd3) hESCs using sequential 3D and 2D cultures have shown spindle morphology similar to fibroblasts. Expression of MSC markers, FIBRONECTIN and CD105, was detected using IF in both Yazd2 (diploid)-MSCs and Yazd3 (triploid)-MSCs. Interestingly, the gene expression profile of MSCs from Yazd2 and Yazd3 hESCs lines were slightly different as Yazd3-MSCs did not express *VIMENTIN* and *CD10*. Also, the expression of VIMENTIN and CD10 was assessed using Western blot analysis, which confirmed the higher expression level of these proteins in Yazd2-MSCs in comparison with Yazd3-MSCs. Decrease of VIMENTIN IFs changes the organization of microfilaments and microtubules, which caused reduced MSCs deformability^[^^17^^]^. On the other hand, reports have shown that CD10 is not an essential marker for MSCs and demonstrated the low expression of this marker in MSCs while strongly expressed in fibroblast^[^^18^^]^. Therefore, these differences in gene expression profile between two hESCs-MSCs lines might be due to the existence of heterogeneous cell populations in Yazd2-MSCs. It is unknown that this discrepancy can be due to the chromosomal differences between the hESC lines or resulted by differentiation process. 

Herein, the expression of specific MSCs markers, including CD105, CD90, CD73, and CD44, using FACS is very similar to other studies^[^^10^^,^^16^^,^^19^^]^, which affirmed the MSCs characteristics of different passage numbers of Yazd2-MSCs. Also, osteogenic differentiation of the hESCs-MSC lines confirmed multipotency and differentiation potential of the MSCs-derived cells from diploid and triploid hESCs.

In conclusion, our study is the first to compare the characteristics of hESCs-MSCs from two different normal (diploid) and abnormal (triploid) pluripotent hESC lines using sequential 3D (embryoid body formation) and 2D (monolayer culture) methods. Therefore*, *3D and 2D culture can be used for the generation of hESC-MSCs from both normal (diploid) and abnormal (triploid) hESCs lines.

. 

However, some differences were observed in their gene expression profiles. According to the therapeutic potential of MSCs, including immunosuppressive feature of the inflammatory disease and tissue transplantation procedure, hESC-MSCs can serve as a clinical source for future human cell-based therapies.
